# Walking the talk in digital transformation of regulatory review

**DOI:** 10.3389/fmed.2023.1233142

**Published:** 2023-07-26

**Authors:** Ramy Khalil, Judith C. Macdonald, Andrew Gustafson, Lina Aljuburi, Fabio Bisordi, Ginny Beakes-Read

**Affiliations:** ^1^Scimitar Inc., Spokane, WA, United States; ^2^Pfizer (United Kingdom), Tadworth, Surrey, United Kingdom; ^3^GlaxoSmithKline (United States), Durham, NC, United States; ^4^Sanofi U.S., Bridgewater, NJ, United States; ^5^Roche (Switzerland), Basel, Switzerland; ^6^Amgen (United States), Thousand Oaks, CA, United States

**Keywords:** cloud, submission and approval, digital transformation and big data, dossier and approval process, structured content, health authorities, data exchange, regulatory review models

## Abstract

Cloud-based regulatory platforms have the potential to substantially transform how regulatory submissions are developed, transmitted, and reviewed across the full life cycle of drug development. The benefits of cloud-based submission and review include accelerating critical therapies to patients in need globally and efficiency gains for both drug developers and regulators. The key challenge is turning the theoretical promise of cloud-based regulatory platforms into reality to further the application of technology in the regulatory processes. In this publication we outline regulatory policy journeys needed to effect the changes in the external environment that would allow for use of a cloud-based technology, discuss the prerequisites to successfully navigate the policy journeys, and elaborate on future possibilities when adoption of cloud-based regulatory technologies is achieved.

## 1. Introduction

Cloud-based regulatory platforms have the potential to substantially transform how regulatory submissions are developed, transmitted, and reviewed across the full life cycle of drug development. The benefits of cloud-based submission and review include accelerating critical therapies to patients in need globally and efficiency gains for both drug developers and regulators (1, 2).

A growing number of regulators have recognized the role that cloud-based approaches can have in their technology plans. These include the FDA Technology and Data Modernization Action Plan (3) and elements related to informatics in the sixth reauthorization of the Prescription Drug User Fee Act (PDUFA VII), as well as the European Union cloud strategy (4). These strategies seek to modernize digital infrastructure to support the respective regulatory networks and create efficiencies in the review process. In addition, regulators have been working to update current review paradigms. In the US the Split Real Time Application Review pilot program (5) allows for a more staged approach to provision of data. Likewise in the EU the draft General Pharmaceutical legislation currently offers the promise of a phased review (6). These constructs do not require a cloud-based platform but could be considerably enhanced by a cloud platform in future.

The key challenge is turning the theoretical promise of cloud-based regulatory platforms into reality, building on the foundation of existing tools – such as CTD and eCTD – to further the application of technology in the regulatory processes.

While the ultimate vision is to provide technology-assisted data analytics and decision-making across the full range of biopharmaceutical product research and development, as well as the pain points associated with post-approval activities (7), we are not recommending any changes to standards of review or the important public health responsibilities that regulators carry out to ensure the safety, efficacy, and quality of medical products.

In this publication we outline regulatory policy journeys needed to effect the changes in the external environment that would allow for use of a cloud-based technology, discuss the prerequisites to successfully navigate the policy journeys, and elaborate on future possibilities when adoption of cloud-based regulatory technologies is achieved.

We describe here the progression along the regulatory policy journeys by describing potential initial cloud-based capabilities and two proofs of concept (POC) from Accumulus Synergy (8),^1^ a developer of one such regulatory solution. The proposed cloud platform aims to facilitate a more dynamic and collaborative review model, which could ultimately support iterative upload of data and dialogue with regulators to improve speed, transparency, and efficiency in the regulatory review and approval process.

**Figure 1:**
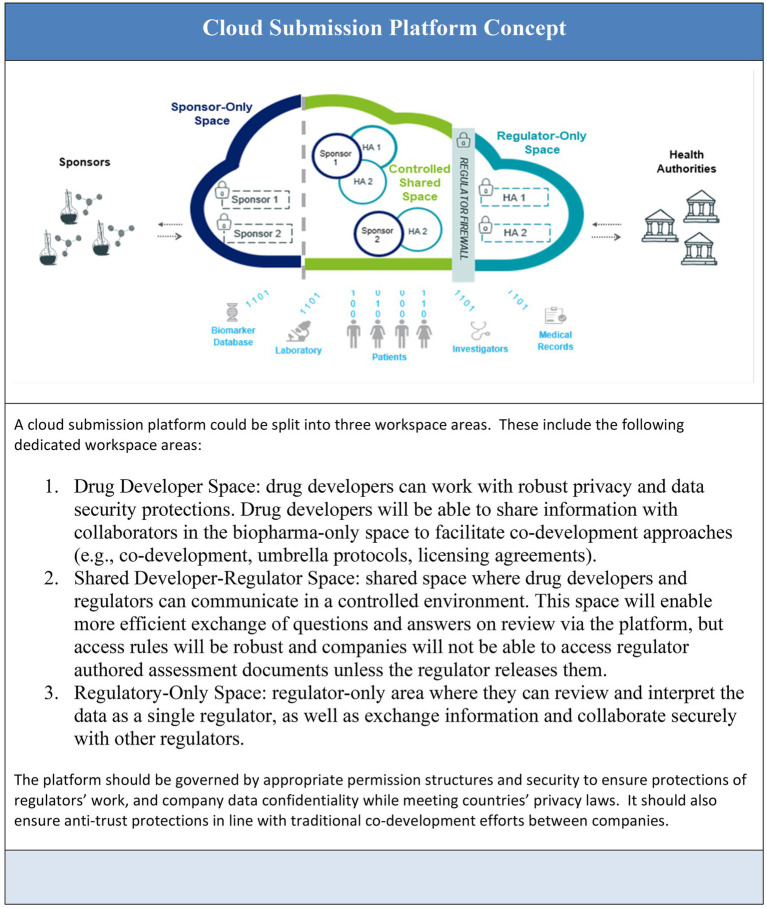
Cloud submission platform concept.

Figure 1 Illustrates the concepts of information flow between submission process participants as the foundation of future state ways of working.

## 2. Regulatory policy journeys

Regulatory policy focuses on evolving the external regulatory environment to support and adopt advances in science, technology, and drug development. A regulatory policy roadmap to articulate the cloud platform vision can be expressed as three major journeys progressing over a multi-year horizon as described in Table 1.

**Table 1:** Policy journey from-to shifts.

Current state	Future state
Journey 1: collaboration via cooperative relationships	
Tendency for siloed individual country submissions by drug developers, in successive waves of priority	Cloud technology facilitates ease of collaboration between regulators leading to greater use of reliance and work-sharing and hence more simultaneous submissions, reviews and approvals, based on common global submission content
Journey 2: efficiency	
Manually intensive PDF document-constrained submissions with resource intensive re-transcription of data hampering trend analysis	Structured data submissions that are both human and machine-readable allowing use of technology for assisted or automated confirmatory re-analysis by regulators
Dialogue with biopharma sponsors generally only at discrete regulator review milestones based on touch points; little to no use of modernized technology assisted regulator reviews	Evolution towards more continuous/iterative data upload and dialogue during review and enriched decision-making
	Continuous data upload with real-time analysis and response
Journey 3: evidence generation, insights, and trends	
Conventional clinical trial data as the primary evidence base with some use of novel sources of evidence	Source-agnostic cloud-platform allows for bringing together diverse types of evidence, (RWD, data from wearables, etc.) alongside traditional sources. Potential new data insights and trends could be unlocked via analysis across unified data pool
Knowledge management is manual, resource intensive and cumbersome resulting in data being used once for a submission and the ability to uncover new insights from data being limited and constrained	More agile knowledge management allowing new insights from data to be uncovered with potential benefits to patients

### 2.1. Journey 1: collaboration *via* cooperative relationships

Cloud-based technology could facilitate scaling of increasing multi-directional collaboration.

Collaboration between regulators is not new. Regulators are already working together on collaborative review processes to promote alignment on identification and resolution of review issues relating to clinical benefit/risk assessments such as FDA’s Project Orbis (9, 10), and to manage their available application review resources more efficiently through work-sharing models such as the Access Consortium (11), or reliance mechanisms such as ZaZiBoNa (12). Benefits to regulators include sharing insights, optimizing resources across multiple organizations, and accelerating therapies that meet approval standards to their patient constituents.

Consolidating the interactions between drug developers and health authorities in a single cloud environment would create a single source of up-to-date referenceable truth for the exchange of information, data, and all aspects of the dialogue including information requests, post market requirements and commitments, and tracking of audit findings. However, this is not something that will be achieved in the short-term across multiple regulators. It still requires a sustained effort on harmonization and convergence of regulatory requirements around the world. A cloud platform removes storage constraints, but care is needed to ensure that this does not inadvertently allow a proliferation of non-value-added administrative documents and bespoke national requirements that do not inform the science of the regulatory review.

Scaling collaboration between multiple regulators or between regulators and drug developers would enable progress along our second proposed regulatory policy journey, efficiency.

### 2.2. Journey 2: efficiency

All regulators and drug developers are challenged by resource constraints including increasing size and complexity of drug development portfolios, the extraordinary scientific advances in recent years which require evolving data generation and review approaches such as FDA’s accelerated approval pathways (13), and continued increase in post-marketing workload demands (7). Biopharma companies also face challenges as they seek to reduce the time it takes to bring products to patients more efficiently (14).

Cloud-based technology platforms offer the ability to realize several efficiencies in regulatory processes, leveraging workflows and optimizing efficiency to benefit public health. Cloud-based solutions will enable greater efficiency of review by providing the opportunity to evolve the dynamics of how review is managed and conducted, for example by allowing automation of routine administrative tasks and freeing up reviewer time for more impactful scientific work (15). Within biopharma companies, cloud-based work coupled with structured content, automation, artificial intelligence such as natural language processing, and machine learning could augment work currently performed by people, including authoring, data analysis, project management, and data/file management (16). All of these proposed changes to ways of working require policy-driven evolution of processes for both regulators and biopharma.

### 2.3. Journey 3: evidence generation, insights, and trends

Historically, randomized controlled clinical trials (RCT) have been the “gold standard” for generating evidence to support biopharma product approvals. While RCT remains the bedrock of risk–benefit decisions, biopharma and regulators are increasingly looking to leverage alternative and scientifically sound sources of clinical data including real world evidence (RWE) sourced from patient registries and electronic health records (17). RWE is becoming more widespread in use, although challenges remain such as heterogeneity, lack of standardization of terms and the large volumes of data. The need for standardization of terminology and heterogeneity of data is not something which will be solved *via* a cloud platform and requires action by appropriate bodies such as ICH. However, a cloud platform is a better storage solution for large volumes of data than today’s submission paradigm.

Clinical trials are also increasingly becoming more digital both in terms of using digital tools to capture the data, (e.g., biosensors/wearables) and by decentralized approaches where the patients participate remotely rather than traveling to study sites (18). Cloud-based regulatory platforms could offer the possibility to house both data from traditional RCTs as well as data from new sources and technologies, allowing potential for integration and analysis across various data types. As observed in the efficiency journey, coupling expanded and unified data sets with artificial intelligence, machine learning, and automation could enable discovery of new trends and insights in appropriate contexts (19). This could be particularly beneficial to regulators when they are examining trends that occur across products from multiple companies.

Evidence generation is evolving, and the use of technology must keep pace in order for data from new sources generation such as biomarkers, digital health tools, medical records, wearables to enhance traditional methods of evidence generation and provide valuable insights that otherwise would not be available. Cloud-based technologies will be necessary to ingest, standardize, exchange, and ultimately analyze the data coming from these new sources.

The journeys, which are over-lapping and interrelated, are a helpful way to envisage the future for regulatory submissions.

## 3. Pre-requisites for progressing the cloud-based regulatory submission and review

To unlock the benefits of cloud-based regulatory submission and review, there are several key policy areas that must be addressed, to create a hospitable operating environment for such cloud platforms, and to help industry navigate across the three journeys described above. These include the establishment of high quality and interoperable data standards, and policies that address data sharing, data privacy, and data security. All these policy areas require broad stakeholder engagement to achieve global scale and ultimately maximum patient benefit (2).

### 3.1. Regulatory harmonization of technical content requirements

Continued and sustained efforts to harmonize technical requirements for regulatory submissions *via* the International Council for Harmonization (ICH) are critical as this helps drive towards common global data requirements (20). Harmonization is a crucial enabler for reliance and work-sharing. Harmonized requirements together with the availability of secure cloud-based platforms can catalyze further collaboration between regulators and enable more patients to benefit from therapies in a timely fashion.

### 3.2. High quality data standards, interoperability, privacy and security

High quality data standards and interoperability are a necessary pre-cursor to support collaboration, streamlined data exchange, and other data driven advancements. Currently, data standards vary widely across regions with some countries only starting to implement digitalization while others have significantly matured their digital health infrastructures. ISO Identification of Medicinal Products (IDMP) specifies standard definitions for the identification and description of medicinal products for human use (21). This will help facilitate the reliable exchange of product information together with data exchange standards such as HL7’s Fast Healthcare Interoperability Resources (FHIR) (22). Both will be critical to the success of any cloud-based platform by harmonizing data standards to ensure interoperability across different geographic regions. It is also essential to ensure that appropriate healthcare data policies are in place that will enable consistent high quality and secure data standards across regions (23).

Interoperability is a necessity for efficient data exchange and a foundational element to any regulator collaboration.^2^ Increasing collaborative reviews, reliance, and work sharing (1) amongst regulators yields efficiency benefits to all participants and serves to reduce global drug approval lag.

Similar to data standardization and interoperability acting as the precursors to the technical exchange of data, policies governing data sharing, data privacy, and data security will also need development and harmonization to address the ethical, political, and patient concerns that could emerge from cloud-based collaboration (24). Policies will be required to address cybersecurity, antitrust/anti-competitive, intellectual property, and other issues (25). Policies and compliance enforcement will also be required to ensure protection of patients and their data (26).

As policies evolve and cascade, existing data infrastructure and agreements between stakeholders for data exchange will need to be re-assessed to ensure that they are fit for purpose.

Regulatory cloud platforms should be designed and built to meet all applicable regional and global privacy laws and implement appropriate safeguards to ensure that all data is protected.

### 3.3. Broad stakeholder engagement

Transitioning the ecosystem to cloud-based platforms is a complex and ambitious endeavor that will require a phased approach to deliver early and focused solutions that can be expanded to achieve the larger potential over an extended time horizon. Successful adoption of cloud-based platforms will ultimately require close partnership, collaboration, and alignment across a large and diverse set of stakeholders [e.g., regulators, drug developers, technology developers, Clinical Research Organizations (CROs)], trade associations, standards organizations (2).

## 4. Cloud platform capabilities

### 4.1. Cloud enabled regulatory collaboration

To realize the vision of cloud-based submission and review, fundamental platform capabilities are required. Two such capabilities – Data Exchange and Submission Review and Collaboration – are key components of the Cloud Platform Concept and detailed below with descriptions of how they relate to the previously outlined policy journey.

#### 4.1.1. Submission review and collaboration

Submission review and collaboration can be developed as a core set of platform capabilities to enable more efficient and secure collaboration between biopharma sponsors, biopharma sponsors and health authorities, or between health authorities. The overall intent is to eliminate traditional document exchange across separate platforms by promoting submission and review in the shared spaces. Working in the shared spaces will reduce unnecessary data handling and transmission while promoting close to real-time exchange of feedback and information.

#### 4.1.2. Data exchange

Cloud-based data exchange capabilities would support a codified, structured, standardized model to streamline data exchange, analysis, and interoperability. The exchange of structured and standardized information between drug developers and regulators could allow drug developers to move away from the current narrative heavy unstructured content and PDF format (Portable Document Format) to transmission of structured source data contained in regulatory filings. A fully digital/cloud-based environment would also require standardization of clinical trial terminology (CDISC) and use of visualization in regulatory review. The platform will need to be able to accommodate this and to also offer regulators tooling to quickly search across the increasing the volume and complexity of the submitted data so that additional data is useful not burdensome.

A more evolved user interface for regulators and drug developers could unite text, graphical data, and source data components into a “single pane of glass” to enhance submission, review, and post-authorization change management *via* optimized data replication, search, and assessment capabilities (27). Such a capability could leverage the latest standards including HL7’s and sit atop a FHIR platform providing a standard for exchanging information across healthcare applications.

Data Exchange capabilities would support submissions that use different data types across the entire drug development lifecycle including pre-clinical, clinical (product safety and efficacy) and chemistry manufacturing and controls (product quality) data as well as evolving to allow for real-time submission and approval as seen in FDA’s pilot, Real-Time Oncology Review or enabling extensions of shelf-life with incrementally new stability data.

### 4.2. Accumulus Synergy proofs of concept: project Orbis and labeling negotiations

Accumulus Synergy is developing a data exchange platform that aims to enable enhanced collaboration and efficiency between life science organizations and global health authorities (8).

Accumulus Synergy will aim to allow regulators and drug developers to road test its cloud platform *via* initial proofs of concept (POCs) and build subsequent learnings into future offerings. The initial offerings are limited in scale and scope to establish proof of concept. Accumulus has identified near-term focus areas for its initial use cases:

1. Project Orbis
2. Labeling negotiations

#### 4.2.1. Project Orbis

Accumulus Synergy is developing a collaboration platform for use in Project Orbis (10), a submission review program initiated by FDA’s Oncology Center of Excellence for concurrent submission review of oncology products among several global health authorities.

Accumulus Synergy’s platform features include:

- Regulatory project creation and management
- Invitation management (GSP [Global Submission Plan] new eForm)
- Document parsing to enable collaboration (AAid [Assessment Aid])
- Novel content editor leveraging structured content for enhanced collaboration
- Project meetings, milestones, and artifact management
- Information request management (regulator questions) and library.

The configurable nature of its cloud capabilities built to support Project Orbis can be re-purposed for other types of collaboration, work-sharing and reliance programs such as ICMRA pilots, ACCESS Consortium, all in support of regulatory harmonization and convergence, worldwide. Remote and hybrid inspections are another tangible example for cloud-based collaboration and opportunity for HAs to adopt Good Reliance Practices (GRelP) (28). The benefits to regulators include sharing insights, optimizing resources across multiple organizations, and accelerating reviews of therapies to their patient constituents.

#### 4.2.2. Labeling negotiations

Labeling negotiations during the marketing application review process showcase the versatility of the Accumulus Synergy platform, applying the collaboration features from its Project Orbis support product to critical regulatory content shared between drug developers and regulators.

Labeling negotiation will leverage previously developed features and functionality:

- Project creation (new project type)
- Document parsing
- Accumulus Synergy’s novel document editor for real time collaboration between the biopharma and a given regulator’s comments, track changes, suggesting edits etc.
- Real time Q and A.

This POC is focused initially on the FDA, but all regulators conduct labeling negotiations with drug developers, so this could be a valuable to additional regulators in the future.

## 5. Discussion

The last decade of digital transformation has driven improvements across industries and across the globe. Digital transformation takes on many familiar forms including cloud-based application access, cloud-based storage, streamlined workflows, improved user experiences, artificial intelligence, and machine learning assisted work.

While stakeholders in the drug development industry have been able to leverage aspects of digital transformation in various parts of the drug development lifecycle, the regulatory framework governing the exchange of information between drug developers and regulators has not fully assimilated technologies available today. This may be due to the complexity of re-imagining the paradigm and the siloed nature of previous attempts (29). At the same time, collaboration between global regulators is growing and reached new levels during the Covid-19 pandemic (30), but this was manual, resource-intensive, and took place on platforms where there were limitations on the types and size of files that could be exchanged. Cloud-based platform capabilities can transform the nature of regulatory data and information exchange. Broad stakeholder engagement to evolve regulatory policies and enable the assimilation of current technologies into today’s regulatory framework could generate substantial benefits for regulators, drug developers, and patients.

The pace of industry evolution will be set by the collective and joint efforts of leading health authorities, drug developers, trade associations, and technology developers. Accumulus Synergy has emerged in response to the need to bring these parties together to address the regulatory framework. With its nonprofit status and focus on global citizens, it is uniquely positioned to develop technologies that can help bridge the needs of drug developers and global regulators. The Accumulus Synergy Platform will aim to validate the cloud and digital transformation hypothesis by first enhancing regulator collaboration mechanisms and then expanding into the exchange of data and information. Over time the aspiration is for the platform to cover all data and information to support regulatory submissions across the drug development lifecycle.

### 5.1. Where are we heading?

An organization such as Accumulus Synergy is needed to generate the activation energy the biopharma industry needs to rally its multiple stakeholders around the possibilities of cloud-based submissions and evolving regulatory frameworks. Such momentum will inspire several trajectories that could be further imagined and explored at the option of regulators and innovators:

1. Expansion within and beyond biopharma to other life sciences sectors
2. Technology Aided and Real-Time Decision Making
3. Expanded Global Collaboration.

#### 5.1.1. Expansion within and beyond biopharma

Once the model of partnership, innovation, and collaboration to shift into cloud-based submissions has been set initially within biopharma, rapid expansion will be needed to support the needs of small and medium sized entities, device and diagnostic providers, and generics. A broader market will also emerge for technology entrants beyond Accumulus Synergy to continuously expand options and improve the industry. The model can scale to support the full remit of health regulators.

#### 5.1.2. Technology aided and real-time decision making

Data standards, interoperability, and security advancements will pave the way for increased use of advanced data analytics, machine learning and artificial intelligence within the regulatory framework (29). Equipping regulators with both data and tools to analyze data rapidly and efficiently at scale could lead to shifts in how their work is performed. Risk-based machine supported, or even automated decision models, will emerge to support regulators with their vast review and decision-making workload.

Additionally, data can be transmitted as it is generated and correspondingly consumed and assimilated into decision models allowing for real-time analysis and decision making versus the current batch model where all the submission data is submitted together after the last component is finalized.

#### 5.1.3. Expanded global collaboration

Increasing the opportunities for technology assisted collaboration creates greater transparency in review and decision making. It will lead to continuous learning, improvement, and innovation within each health authority, and possibly sharing of practices. Levels of collaboration could be achieved where both work and decisions are shared, and greater levels of reliance and possibly convergence could be achieved, bringing the greatest acceleration value to patients as more global citizens could benefit from concurrent decisions around therapeutic safety, efficacy, and quality.

## 6. Conclusion

Re-designing the paradigm from a document centric mindset to a data centric approach is a bold, transformative, multi-year endeavor and will ultimately touch all aspects of research, development and life cycle management. Journeying towards this will unlock efficiencies not yet available to drug developers and regulatory authorities. There are many practical aspects of this new paradigm to be worked out which are beyond the scope of this short paper. We call drug developers, regulators, trade associations and other key stakeholders to work together in supporting harmonized efforts towards the development of cloud-based technologies that will drive greater industry productivity, acceleration, and patient benefit.

## Data availability statement

The original contributions presented in the study are included in the article/supplementary material, further inquiries can be directed to the corresponding author.

## Author contributions

All authors listed have made a substantial, direct, and intellectual contribution to the work, and approved it for publication.

## Conflict of interest

RK was employed by Scimitar Inc. JM was employed by Pfizer Inc. AG was employed by GlaxoSmithKline. LA was employed by Sanofi. FB was employed by F. Hoffmann-La Roche Ltd. GB-R was employed by Amgen, Inc.

## Publisher’s note

All claims expressed in this article are solely those of the authors and do not necessarily represent those of their affiliated organizations, or those of the publisher, the editors and the reviewers. Any product that may be evaluated in this article, or claim that may be made by its manufacturer, is not guaranteed or endorsed by the publisher.

## Footnotes

^1^Accumulus Synergy Inc. was formed in 2020 as a non-profit trade association advocating for digital transformation and regulatory harmonization. The company is developing a data exchange platform to enable enhanced collaboration and efficiency between life sciences organizations and health authorities worldwide. It has secured sponsorship from 12 leading biopharmas including Amgen, Astellas, AstraZeneca, Bristol Myers Squibb, Eli Lilly, GlaxoSmithKline, Johnson & Johnson, Merck, Pfizer, Roche/Genentech, Sanofi, and Takeda.

^2^Draft *CF* Reg. Data policy paper.
